# Localization of accessory pathways in Wolff‐Parkinson‐white syndrome using ECG‐based multi‐task deep learning

**DOI:** 10.1111/eci.14385

**Published:** 2025-04-07

**Authors:** Jasper Hennecken, Bauke K. O. Arends, Thomas Mast, Lukas Dekker, Pim van der Harst, Yuri Blaauw, Wolfgang Dichtl, Thomas Senoner, Rutger J. Hassink, Peter Loh, René van Es, Rutger R. van de Leur

**Affiliations:** ^1^ Department of Cardiology University Medical Centre Utrecht Utrecht The Netherlands; ^2^ Department of Cardiology Catharina Hospital Eindhoven Eindhoven The Netherlands; ^3^ Department of Cardiology University Medical Centre Groningen Groningen The Netherlands; ^4^ Department of Cardiology Innsbruck Medical University Innsbruck Austria; ^5^ Department of Anesthesiology and Intensive Care Innsbruck Medical University Innsbruck Austria

**Keywords:** accessory pathway location, deep learning, electrocardiogram, multi‐task learning, Wolff‐Parkinson‐white

## Abstract

**Background:**

Wolff‐Parkinson‐White syndrome is characterized by accessory atrioventricular pathways (AP) and atrio‐ventricular re‐entry arrhythmias. Catheter ablation approach and success are determined by AP location. Existing rule‐based algorithms based on the electrocardiogram (ECG) are time consuming, prone to inter‐observer variability and use delta wave polarity as a binary variable. To overcome these challenges, we propose a model based on a deep neural network (DNN).

**Methods:**

Patients with concealed pathways, multiple antegrade conducting pathways or without any sinus rhythm ECGs were excluded. AP location was determined based on electrophysiological testing during catheter ablation and categorized into right‐sided, septal and left‐sided APs. Multi‐task learning with auxiliary identification of the presence of pre‐excitation, parahisian pathways and locations where a transseptal puncture is potentially required was used to increase usability and performance. The DNN was compared to the Milstein and Arruda algorithms.

**Results:**

Between 1997 and 2023, 645 patients who underwent catheter ablation for an AP were included in the study. The model was developed using 1.394 ECGs from 567 patients. The DNN was tested using 78 ECGs in two independent cohorts. The model outperformed both the Milstein and Arruda algorithms with an area under the receiver operating characteristic curve (AUROC) of .92 (95% confidence interval: .88–.96) compared to the Arruda algorithm (AUROC of .80; *p* <.001) and the Milstein algorithm (AUROC of .81; *p* <.001).

**Conclusions:**

Our model showed excellent discriminatory performance in predicting the location of an accessory pathway while outperforming conventional techniques. Clinically, this tool can improve preoperative planning and risk stratification.

## Graphical abstract

Annulus fibrosus of the heart, illustrating the discriminatory performance of the deep neural network in differentiating right‐sided, septal, and left‐sided accessory pathways. area under the receiver operating curve (AUROC), sensitivity (SENS), specificity (SPEC).

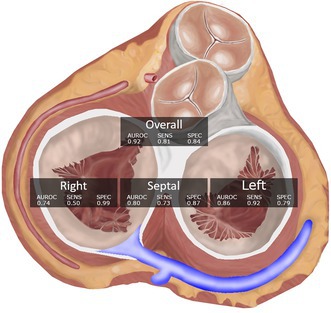

## 1 INTRODUCTION

Wolff‐Parkinson‐White (WPW) syndrome is a congenital heart disease that arises from aberrant conduction over an accessory atrioventricular pathway (AP).^1^, ^2^, ^3^, ^4^, ^5^, ^6^, ^7^, ^8^, ^9^ Consequently, WPW predisposes to paroxysmal supraventricular tachycardias based on either re‐entry over the AV node and the AP or atrial arrhythmias descending through the bypass tract.^1^, ^2^, ^3^, ^4^, ^5^ Patients are usually asymptomatic but can experience palpitations, lightheadedness and pre‐syncope due to reciprocating atrioventricular reentrant tachycardias.^1^, ^2^, ^3^, ^4^, ^5^ Although rare, atrial fibrillation rapidly conducted over a malignant conducting AP can trigger ventricular fibrillation resulting in sudden cardiac death.^1^, ^2^, ^3^, ^4^ The pre‐excitation in WPW syndrome is characterized by specific features on the electrocardiogram (ECG): a short PR interval, slurred upstroke of the QRS (‘delta wave’) and a wide QRS.^3^, ^4^, ^5^ This WPW pattern can be seen in up to .25% of the general population and can be intermittent or even permanently disappear (in up to 40% of patients) over time.^3^, ^4^ Current European guidelines recommend catheter ablation for both patients with symptomatic‐ and asymptomatic WPW syndrome.^3^, ^4^

The location of the AP severely influences the difficulty of the catheter ablation and subsequently its effectiveness.^3^, ^6^, ^7^, ^8^, ^9^ Patients with a parahisian AP, for example, are at risk for atrioventricular blocks and have a higher recurrence risk of pre‐excitation.^6^, ^7^, ^8^ Ablation of deep myocardial paraseptal APs is challenging and often requires multiple ablation sessions from both the right and left sides as well as coronary sinus applications.^1^, ^7^, ^8^ APs within the left free wall can be accessed via either a retrograde approach through the aorta or antegrade using a transseptal puncture.^7^, ^8^, ^9^ Although complications are rare, a transseptal puncture is technically demanding and requires a sound understanding of atrial anatomy whereas a retrograde aortic approach is time‐consuming and carries additional vascular complication risks.^7^, ^8^, ^9^ Pre‐procedural knowledge of the AP location could allow for better planning, faster procedures, as well as decreased exposure to ionizing radiation and unnecessary transseptal punctures, allowing an early choice of appropriate catheters and energy sources.^7^, ^8^

There are several manual decision tree algorithms that rely on a stepwise classification of delta wave polarity in coupled leads for prediction of AP location.^10^, ^11^, ^12^, ^13^ Minor disagreements between observers over QRS transition point or delta wave axis can easily lead to different classifications due to the dichotomous nature of these algorithms.^10^, ^11^ The analysis of pathway location using these stepwise algorithms is prone to inter‐observer variability and has an overall low predictive accuracy.^10^, ^11^ Deep neural networks (DNNs) can extract patterns from complex signals, such as ECG data,^14^, ^15^, ^16^ and use the entire signal as input, rather than using extracted features.^14^ This type of analysis is automatic, efficient and reduces the time and effort required for analysis.^14^, ^15^, ^16^ DNNs can be trained using multi‐task learning, thereby performing auxiliary tasks with distinct noise patterns introducing an inductive transfer, guiding the model to favour certain hypotheses and reducing its ability to fit on random noise.^17^ By learning different tasks with distinct noise patterns, the model builds more generalized and robust representations.^17^

We propose a DNN‐based tool with multi‐task learning that classifies pathway locations (right, septal, and left) while also predicting the presence of pre‐excitation, identifying AP locations potentially requiring a transseptal puncture, and detecting parahisian pathways.

## 2 METHODS

### 2.1 Data acquisition

All data was obtained from databases at the University Medical Centre Utrecht (UMCU), University Medical Centre Groningen (UMCG), the Catharina Hospital Eindhoven (CZE) and the Medical University of Innsbruck (MUI). We extracted median beat waveforms from the raw 10‐s 12‐lead ECG using the generic algorithm from the MUSE ECG system (MUSE version 8; GE Healthcare). ECGs acquired at 250 Hz were resampled to 500 Hz. All extracted data was anonymized in accordance with the EU General Data Protection Regulation.

### 2.2 Patient identification

**FIGURE 1:**
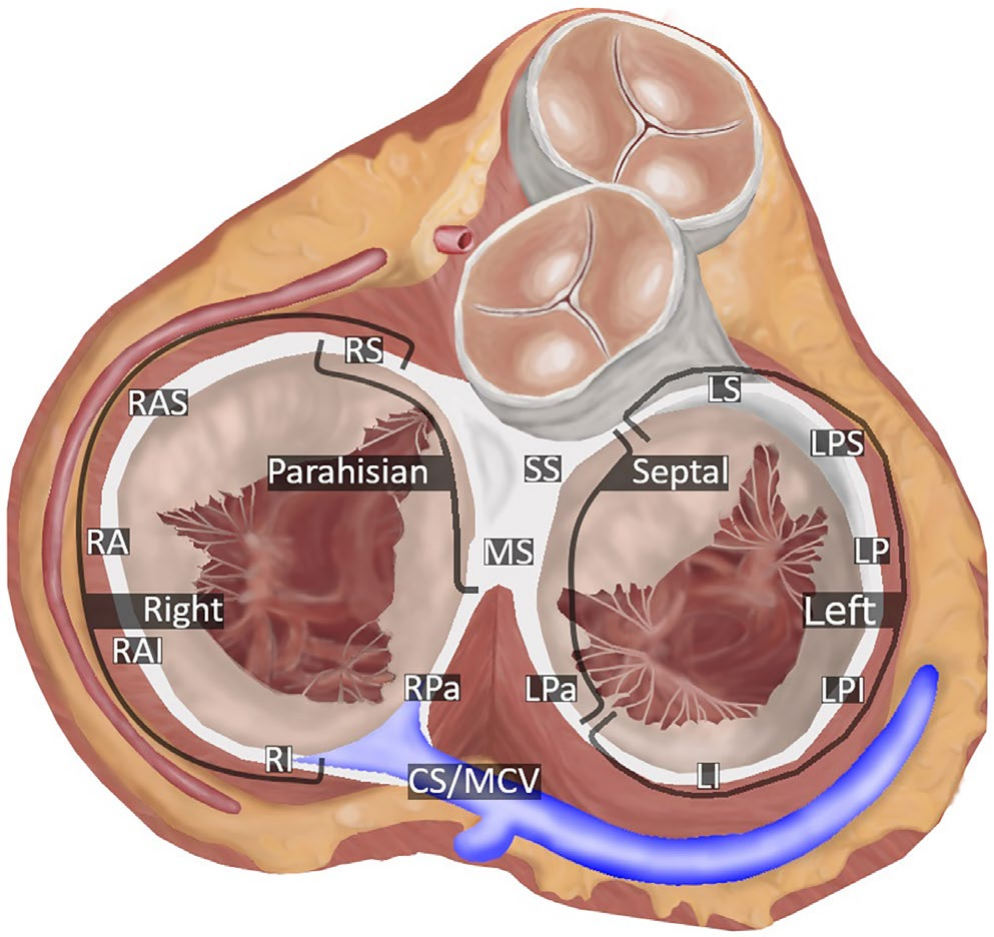
Annulus fibrosus of the heart with respective pathway locations described by Cosio et al.^18^ Right pathways: Right Superior (RS), Right Anterosuperior (RAS), Right Anterior (RA), Right Anteroinferior (RAI), Right Inferior (RI). Septal pathways: Superoseptal (SS), Midseptal (MS), Left Paraseptal (LPa), Right Paraseptal (RPa), Coronary Sinus (CS), Middle Cardiac Vein (MCV). Left pathways: Left Superior (LS), Left Posterosuperior (LPS), Left Posterior (LP), Left Posteroinferior (LPI), Left Inferior (LI). Parahisian pathways: Right Superior, Superoseptal, Midseptal.

Patients that underwent electrophysiological testing at the UMCU, the UMCG, CZE or the MUI were identified. Basic patient information was extracted from the patient records. Date of the ablation, number of pathways found, AP location and conduction parameters were extracted from the electrophysiological testing report and evaluated. If outdated terminology was used to describe AP location, conversion to anatomically correct locations described by Cosio et al^18^ was made. A schematic view of all anatomically correct locations around the annulus fibrosus can be seen in Figure 1. Patients with WPW were included if they received catheter ablation. Exclusion criteria were multiple antegrade conducting pathways, no clear pathway location description, concealed pathways with only retrograde conduction or no electrophysiological testing report found.

### 2.3 Data curation and annotation

ECGs were included if they were made before the first catheter ablation and subsequently assessed by one investigator. ECGs of poor quality or containing significant artefacts (e.g. baseline wander, noise) were excluded. ECGs containing no significant pre‐excitation (no shortened PR interval and no presence of a delta waves) were excluded. ECGs with dubious delta waves, borderline PR interval or right anterior pathways (due to concurrent locations between Cosio et al^18^ and the outdated terminology) were evaluated in conjunction with an electrophysiologist. External data from the MUI^15^ test cohort was additionally screened by two independent investigators on presence of pre‐excitation and conflicting cases were resolved within the study group. An AP was considered parahisian if the AP was in proximity to the bundle of His and was either determined to be superoseptal, right superior or mid‐septal. AP locations were classified as potentially requiring a transseptal puncture if it was a left sided bundle, including left paraseptal pathways.

To further analyse the clinical application of the DNN, the performance of the model was compared to the stepwise algorithms Milstein^12^ and Arruda.^13^ Predictions of these algorithm were constructed by one investigator using the full 10‐s 12‐lead ECG dataset from both the UMCU and the UMCG cohorts, blinded to any prior knowledge of the AP location. Arruda's 13 AP locations were consolidated into right‐sided, septal and left‐sided pathway locations to enable a balanced comparison with the DNN's classifications.

### 2.4 Matching

To adequately identify pre‐excitation, ECGs containing pre‐excitation were matched with control ECGs. Pre‐excitation ECGs were matched 1:1 within the training cohorts and 1:400 within the validation‐ and testing cohorts in accordance with the real‐world incidence of WPW^4^. Matching was done randomly within the UMCG cohort and with propensity scores based on age and sex within both the retrospective UMCU/CZE and prospective UMCU cohorts. All ECGs of the initially included WPW patients were excluded from the control cohorts. One ECG per control patient was used.

### 2.5 Network architecture

**FIGURE 2:**
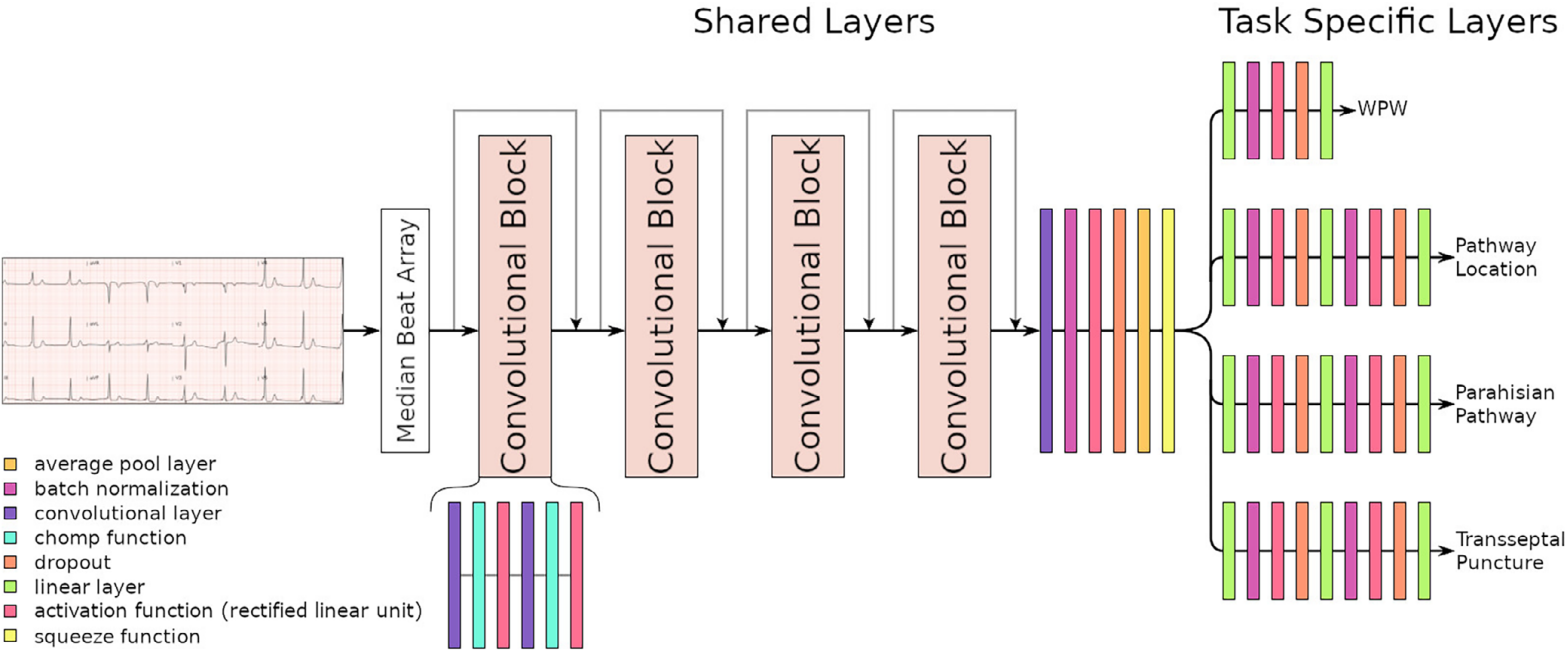
Overview of the network architecture. Description: 4 convolutional blocks followed by a split into linear fully connected layers for each consecutive task. Wolff‐Parkinson‐White syndrome (WPW).

In this paper we use a causal convolutional neural network adapted from van de Leur et al^14^ with multi‐task learning. By sharing representations across tasks, the model can leverage additional information, improving overall performance and preventing overfitting.^16^ The dilatation in the causal convolutions contribute to a large receptive field at the cost of sparsity making an efficient and robust model. After 4 dilated causal convolutional blocks, the squeeze operation collapses the output to a lower complexity. This transformation ensures consistency for downstream processing, where the main shared layers diverge into separate branches of fully connected linear layers, each tailored to a specific task. The branches subsequently have an adaptive amount of fully connected linear layers after which they finally form each respective output‐layer. The number of fully connected linear layers was approximated using the similarity and complexity of the estimated features desired for each consecutive task. Cross‐entropy was used to calculate losses independently for each consecutive branch. Weights were assigned based on their approximated prevalence within the dataset. The model was optimized using the Adam optimizer and a batch size of 128. A rectified linear unit was used as activation function. An overview of the network architecture is given in Figure 2.

### 2.6 Training

Training and validation cohorts were constructed from derived data from the UMCU, the UMCG and the CZE. The model was trained using federated learning between the UMCU/CZE cohort and the UMCG cohort due to privacy concerns. The training data was processed on internal servers, while model parameters were transferred via a secure cloud infrastructure to ensure data confidentiality. Fine‐tuning of the task specific layers of the pathway location, parahisian pathway and the transseptal puncture tasks were further optimized due to class imbalances and complexity of the tasks. All in all, a total of 30 epochs was subdivided into 10 regular epochs with a learning rate of .0005 and fine‐tuning of 5 epochs with a learning rate of .0001 in the UMCG and the UMCU/CZE consecutively. To improve convergence and enhance generalization a Cosine Annealing learning rate scheduler with Tmax of 2500 was used.

### 2.7 Testing and statistical analysis

Performance of the DNN was evaluated using an internal prospective testing cohort with ECG data from the UMCU dating from 2021 to 2023 and an external testing cohort from the MUI.^15^ Splits were made between patients, all patients in the training and validation cohorts were excluded from the prospective testing cohort and one ECG per patient was used. All secondary outcomes were calculated regardless of successful identification of pre‐excitation. Optimal thresholds were approximated using Cohen's Kappa statistic between the cumulative testing cohorts. Bootstrapped results were calculated over 2000 iterations. A stratified split was used for multiclass classification due to severe class imbalances. Area under the receiver operating characteristic curve (AUROC) for multiclass classification was calculated using a weighted one‐vs‐one approach. Comparative analysis between the DNN and the stepwise algorithms was done using the De‐Long test. Non‐inferiority was calculated using a margin of 10%. Continuous variables are expressed as means ± standard deviation. Categorical variables are expressed as absolute values and percentages. Statistical analyses were performed using Python 3.10.2.

## 3 RESULTS

**FIGURE 3:**
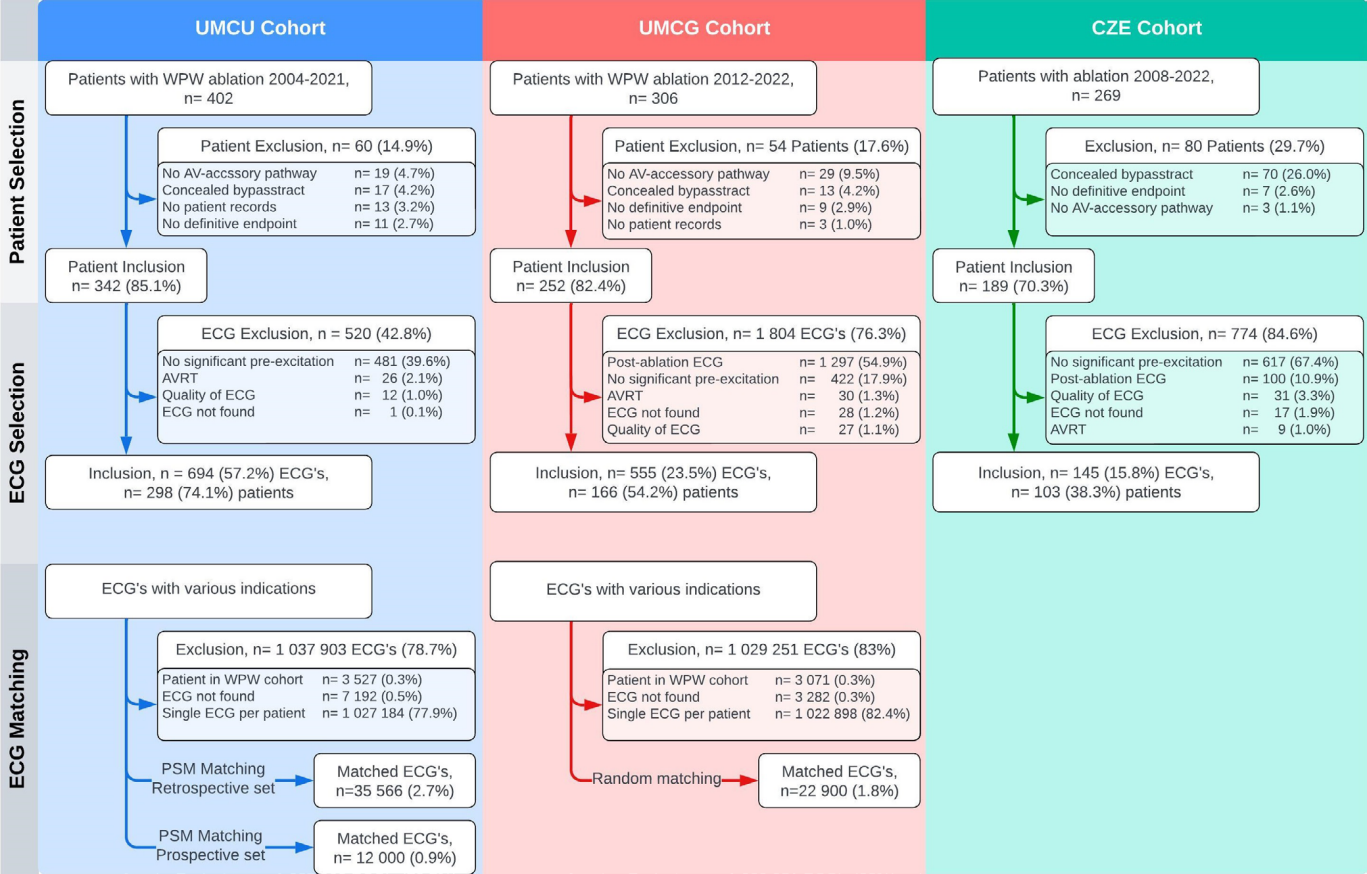
Flow diagram of the training, control and validation cohorts. University Medical Centre Utrecht (UMCU), University Medical Centre Groningen (UMCG), Catharina Hospital Eindhoven (CZE), Wolff‐Parkinson‐White syndrome (WPW), atrio‐ventricular (AV), electrocardiogram (ECG), atrioventricular re‐entry tachycardia (AVRT).

**FIGURE 4:**
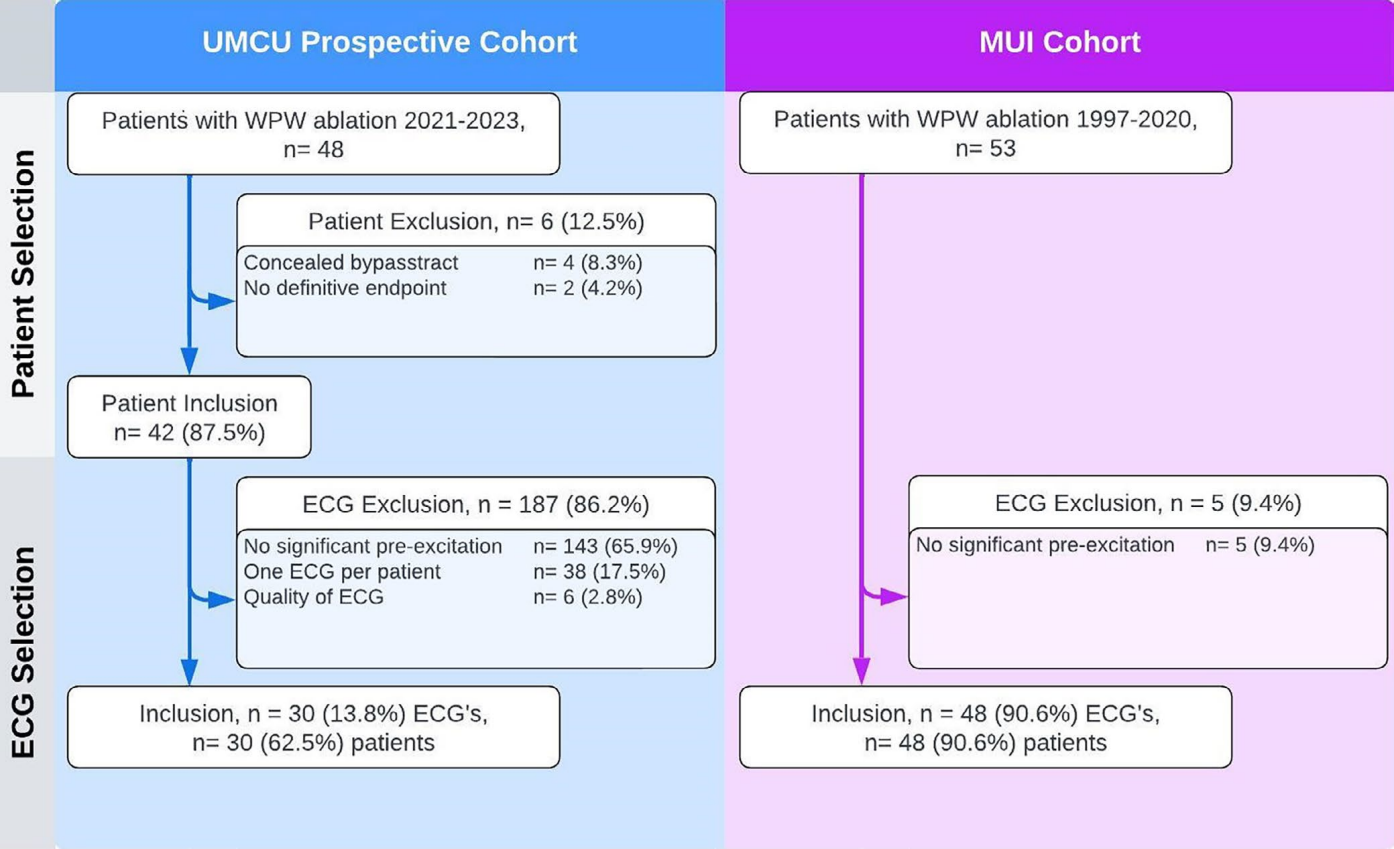
Flow diagram of the testing cohorts. University Medical Centre Utrecht (UMCU), Medical University Innsbruck (MUI), Wolff‐Parkinson‐White syndrome (WPW), electrocardiogram (ECG).

A total of 977 patients were initially identified and 1472 ECGs of 645 patients were included within this study. Pre‐excitation ECGs from the retrospective UMCU database, prospective UMCU database and the UMCG database were matched with 35,566, 12,000 and 22,900 ECGs respectively. An elaborate description of the inclusion and exclusion within the training, validation and control cohorts can be found in Figure 3. A total of 78 patients were used for testing the model. Fifty one patients were initially identified within the UMCU prospective testing cohort, and 30 patients were eventually included. The external testing cohort from the MUI consisted of 53 ECGs of which 5 ECGs were excluded. An elaborate description of the inclusion and exclusion of the testing cohorts can be found within Figure 4.

Overall, the mean age at ablation was 34.8 ± 16.7 years, and 408 patients (63.3%) were male. Additional concealed pathways were prevalent in 27 patients (4.2%) and 5 patients (.8%) were known to have Ebstein's anomaly. Left posterior pathways were most prevalent with 29.6% followed by right paraseptal, left paraseptal and superoseptal with 16.3%, 7.8% and 7.6% respectively. Ninety‐nine patients (15.3%) had a parahisian pathway and 369 (57.2%) had an AP location potentially requiring a transseptal puncture. The mean PR interval was 114.0 ± 20.7 ms. Patient characteristics of all the cohorts can be viewed in Table 1.

**TABLE 1:** Baseline table.

		Overall	UMCU‐R	UMCG	CZE	MUI	UMCU‐P
*n*		645	298	166	103	48	30
Age, mean (SD)		34.8 (16.7)	35.0 (16.4)	30.2 (17.9)	40.1 (15.7)	29.6 (14.8)	35.3 (17.8)
Gender (male), *n* (%)		408 (63.3)	200 (67.1)	91 (54.8)	61 (59.2)	34 (70.8)	22 (73.3)
Location, *n* (%)							
Right	Overall	49 (7.7)	22 (7.3)	17 (10.2)	4 (3.9)	5 (10.5)	1 (3.3)
	Superior	10 (1.6)	4 (1.3)	4 (2.4)	0 (.0)	1 (2.1)	1 (3.3)
	Anterosuperior	11 (1.7)	3 (1.0)	6 (3.6)	0 (.0)	2 (4.2)	0 (.0)
	Anterior	9 (1.4)	3 (1.0)	3 (1.8)	2 (1.9)	1 (2.1)	0 (.0)
	Anteroinferior	5 (.8)	3 (1.0)	1 (.6)	1 (1.0)	0 (.0)	0 (.0)
	Inferior	14 (2.2)	9 (3.0)	3 (1.8)	1 (1.0)	1 (2.1)	0 (.0)
Septal	Overall	277 (43.0)	121 (4.6)	82 (49.3)	41 (39.9)	23 (48.0)	10 (33.3)
	Superoseptal	49 (7.6)	29 (9.7)	11 (6.6)	5 (4.9)	2 (4.2)	2 (6.7)
	Mid‐septal	40 (6.2)	11 (3.7)	13 (7.8)	7 (6.8)	8 (16.7)	1 (3.3)
	CS/MCV	31 (4.8)	10 (3.4)	17 (10.2)	3 (2.9)	0 (.0)	1 (3.3)
	Right paraseptal	105 (16.3)	53 (17.8)	24 (14.5)	15 (14.6)	10 (20.8)	3 (10.0)
	Left paraseptal	50 (7.8)	18 (6.0)	17 (10.2)	11 (10.7)	1 (2.1)	3 (10.0)
	Paraseptal unspecified	2 (.3)	0 (.0)	0 (.0)	0 (.0)	2 (4.2)	0 (.0)
Left	Overall	319 (49.4)	155 (52.0)	67 (40.3)	58 (56.2)	20 (41.6)	19 (63.4)
	Superior	13 (2.0)	5 (1.7)	6 (3.6)	0 (.0)	1 (2.1)	1 (3.3)
	Posterosuperior	48 (7.4)	27 (9.1)	8 (4.8)	7 (6.8)	3 (6.2)	3 (10.0)
	Posterior	191 (29.6)	78 (26.2)	48 (28.9)	43 (41.7)	11 (22.9)	11 (36.7)
	Posteroinferior	43 (6.7)	32 (10.7)	4 (2.4)	3 (2.9)	2 (4.2)	2 (6.7)
	Inferior	20 (3.1)	12 (4.0)	1 (.6)	2 (1.9)	3 (6.2)	2 (6.7)
	Left unspecified	4 (.6)	1 (.3)	0 (.0)	3 (2.9)	0 (.0)	0 (.0)
Parahisian pathway, *n* (%)		99 (15.3)	44 (14.8)	28 (16.9)	12 (11.7)	11 (22.9)	4 (13.3)
Potential TSP, *n* (%)		369 (57.2)	173 (58.1)	84 (50.6)	69 (67.0)	21 (43.8)	22 (73.3)
Multiple pathways, *n* (%)		27 (4.2)	19 (6.4)	3 (1.8)	2 (1.9)	N/A	3 (10.0)
Morbus Ebstein, *n* (%)		5 (.8)	4 (1.3)	1 (.6)	0 (.0)	N/A	0 (.0)

Abbreviations: CS, Coronary sinus; CZE, Catharina Hospital Eindhoven; MCV, Middle Cardiac Vein; MUI, Medical University Innsbruck; NA, Not Available; P, Prospective; R, Retrospective; TSSP, Transseptal Puncture; UMCG, University Medical Centre Groningen; UMCU, University Medical Centre Utrecht.

The DNN showed excellent classification of AP location within the three regions (right, septal and left) and yielded an AUROC of .98 (95% CI: .95–1.00) and accuracy of .86 (95% CI: .74–.96) within the prospective UMCU test cohort. Model performance decreased slightly when tested on the external MUI cohort, yielding an AUROC of .87 (95% CI: .81–.93) and accuracy of .83 (95% CI: .74–.92).

Overall, when combining both testing cohorts, the model yielded an AUROC of .92 (95% CI: .88–.96), sensitivity of .81 (95% CI: .73–.88), a specificity of .84 (95% CI: .77–.91) and an accuracy of .84 (95% CI: .77–.91). Right‐sided pathways demonstrated inferior outcomes compared to septal and left‐sided pathways (right‐sided: AUROC .74, sensitivity .50, specificity .99, septal: AUROC .80, sensitivity .73, specificity .87 and left‐sided: AUROC .86, sensitivity .92, specificity .79). A detailed overview of the performance can be viewed in Table S3.

The model identified pre‐excitation with an AUROC of .96 (95% confidence interval (95% CI): .89–1.00), sensitivity of .57 (95% CI: .39–.75) and a specificity of 1.00 (95% CI: 1.00–1.00). Although the negative predictive value was 1.00 (95% CI: 1.00–1.00), the positive predictive value was .39 (95% CI: .24–.53).

Parahisian pathway identification yielded AUROCs of .95 (95% CI: .84–1.00) and .83 (95% CI: .69–.93) within the prospective UMCU and the external MUI testing cohorts, respectively. The DNN identified locations potentially requiring a transseptal puncture better within the UMCU testing cohort (AUROC .93, accuracy .83) compared to the MUI testing cohort (AUROC .90, accuracy .83). All outcome metrics (AUROC, sensitivity, specificity, negative predictive value, positive predictive value and accuracy) of both testing cohorts can be seen within the supplementary material.

**FIGURE 5:**
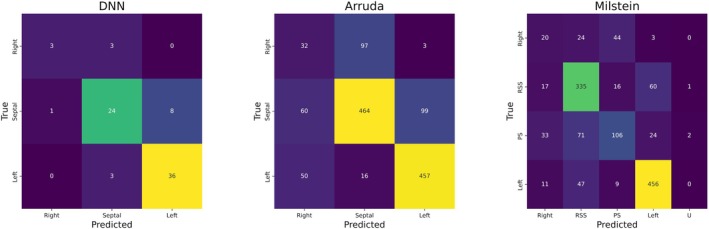
Confusion matrices of the pooled performance of the DNN, merged Arruda and the Milstein algorithm. Description: Predictions of right sided, septal and left sided pathway for the DNN and Arruda. Right sided, right superoseptal (RSS), paraseptal (PS), left sided and undefined (U) pathway location prediction for Milstein. Deep neural network (DNN), right superoseptal (RSS), paraseptal (PS), undefined (U).

The Milstein algorithm yielded an AUROC of .81 (95% CI: .80–.83) and accuracy of .85 (95% CI: .84–.86). The merged Arruda algorithm performed significantly better than the standard Arruda algorithm (AUROC of .80 vs.71 respectively) and yielded similar results compared to the Milstein algorithm (AUROC of .81 vs.80 *p* = .665). The combined testing cohort (of the UMCU‐ and the MUI test cohort) yielded an AUROC of .92 (95% CI: .88–.96) and accuracy of .84 (95% CI: .77–.91), outperforming both the Milstein‐ (AUROC .92 vs.81, *p* <.001) and the Arruda algorithm (AUROC .92 vs.80, p <.001). Performance of the pooled DNN, Milstein and Arruda algorithms can be found in Table 2, confusion matrices can be seen in Figure 5.

**TABLE 2:** Accessory pathway location prediction.

AP location prediction		AUROC	Sens	Spec	PPV	NPV	Accuracy
DNN, value [95% CI]		.92 [.88–.96]	.81 [.73–.88]	.84 [.77–.91]	.81 [.72–.89]	.87 [.81–.93]	.84 [.77–.91]
Milstein, value [95% CI]		.81 [.80–.83]	.72 [.70–.74]	.88 [.87–.89]	.71 [.69–.73]	.90 [.89–.91]	.85 [.84–.86]
Arruda	Raw, value [95% CI]	.71 [.70–.72]	.48 [.46–.50]	.93 [.93–.94]	.49 [.47–.52]	.92 [.91–.93]	.48 [.46–.51]
	Merged, value [95% CI]	.80 [.79–.82]	.74 [.72–.76]	.84 [.83–.86]	.74 [.72–.76]	.83 [.82–.85]	.82 [.80–.83]

*Note*: Table 2 description: performance of the DNN on the combined testing cohort's vs. the conventional methods on the training data. Area under the receiver operating characteristic curve (AUROC), sensitivity (Sens), specificity (Spec), positive predictive value (PPV), negative predictive value (NPV), 95% confidence interval (95% CI), deep neural network (DNN).

## 4 DISCUSSION

In the current study, we developed a multi‐task DNN to predict the most important clinical questions around patients with WPW. The model showed excellent performance in predicting AP location while additionally identifying pre‐excitation, predicting the presence of parahisian pathways and locations potentially requiring a transseptal puncture. Multi‐task learning was used to prevent overfitting and as our algorithm produces multiple predictions using only one input, it is more efficient with reduced computational cost. Our model could provide an efficient and automatic solution for detection of pre‐excitation, better planning, faster procedures, as well as decreased exposure to ionizing radiation. Prediction of parahisian pathways and pathways potentially requiring a transseptal puncture allow for better weighing the risks of complications and appropriate catheter selection.

Our DNN outperformed the conventional stepwise methods of the Milstein (AUROC .92 vs.81, *p* <.001) and Arruda (AUROC .92 vs.71, *p* <.001) algorithms. The original Arruda algorithm has a more detailed prediction of the anatomical location around the annulus fibrosus. To compensate for this, we tested the DNN against a merged Arruda algorithm classifying into right‐sided, septal and left‐sided locations. The DNN showed robust results against the merged Arruda algorithm showing non‐inferiority (.92 vs. 81 *p* <.001).

Previous research on deep learning solutions for AP location prediction^15^, ^16^ is predominantly based on small cohorts with 206–250 patients and is therefore very prone to overfitting on the dataset. Furthermore, Nishimori et al^16^ uses imaging data to further increase performance, decreasing its applicability in clinical workflow when there is no imaging data available. Additionally, the model by Senoner et al^15^ incorporated all locations on the annulus fibrosus, enhancing its specificity. However, several low‐prevalence locations had a sensitivity of 0 during testing, indicating that the model was unable to predict these fine‐grained locations.

We developed our DNN using the largest cohort to date with 567 patients, increasing the performance of the DNN with an overall accuracy of .84 (95% CI: .77–.91) compared to previous work from Senoner et al^15^ with an accuracy of .77 and Nishimori et al^16^ with a mean accuracy of .78 and .8.

Sub‐analysis of both testing cohorts showed a divergence between discriminatory performance of right‐sided AP locations with the UMCU prospective testing cohort (AUROC .98, accuracy .97) and the external MUI testing cohort (AUROC .60, accuracy .92). This difference is possibly due to scarcity within the UMCU cohort as there was only one right‐sided pathway included. Additionally, recognition of pre‐excitation was relatively low with a sensitivity .57 (95% CI: .39–.75). This could be due to the training scheme with fine‐tuning training epochs on the tasks without the identification of pre‐excitation. Development of algorithms with data acquired from the general population would yield more representable results and increase the clinical applicability.

Furthermore, we could not adjust the Milstein algorithm to properly align with the DNN's classification system (right‐sided, septal, and left‐sided APs) for a balanced comparison. The Milstein algorithm contains five prediction classes, including an undetermined pathway location and right superoseptal pathways which cannot be classified into either right‐sided or septal locations.

We tried to predict malignant antegrade conduction properties based on the ECG and clinical characteristics as an auxiliary task. Unfortunately, we did not attain satisfactory performance and thus excluded it from the model. In the future, it may become possible to predict malignant pathways as we deepen our understanding of the underlying etiological factors of their malignancy.

For the development of future algorithms, inclusion of patients with multiple antegrade conducting APs and ECGs with grave artefacts could increase robustness and clinical applicability. Algorithms using multimodal data can further increase performance and robustness. Nishimori et al^16^ showed an increase in AP localization performance when adding chest X‐rays to the ECG data. Apostolopoulos et al^19^ showed that the prediction of coronary artery disease yielded better results using a multimodal neural network with clinical data, myocardial perfusion imaging and coronary angiography. A recent study^20^ showed that 2D speckle‐tracking echocardiography can be useful to predict ventricular activation patterns and therefore AP location. Moreover, deep learning algorithms can benefit (much like human learning) from multiple types of output and input.^17^

## 5 CONCLUSION

We propose a DNN to predict AP location in WPW, addressing limitations of existing conventional stepwise algorithms. The model was developed using 1394 ECGs from 567 patients and optimized using multi‐task learning. The DNN achieved an AUROC of .92, significantly outperforming the Milstein (AUROC .81; *p* <.01) and Arruda (AUROC .80; *p* <.01) algorithms. This innovative tool enhances pre‐procedural planning and risk stratification for catheter ablation, demonstrating superior performance and robustness in localizing APs compared to conventional methods.

## AUTHOR CONTRIBUTIONS

J.H.: contributed to the conception and design of the study, contributed to data acquisition, selected and reviewed the acquired data, adapted the existing algorithm from RvdL and RvE, computationally analysed results and wrote the final manuscript. RvdL: took a leading role equal to RvE in the conception and design of the study, took a leading role in coordinating data acquisition through different institutions, took a supervising role regarding the analysis, and critically reviewed the manuscript for important intellectual content. RvE: took a leading role in the conception and design of the study, took a leading role in the acquisition of funding for the study, and critically reviewed the manuscript for important intellectual content. B.A: assisted in data review, critically reviewed the manuscript for important intellectual content. P.L: took a supervising role in the data analysis, assisted in the conception and design of the study and reviewed the manuscript for editing. T.M, Y.B, L.D, W.D: took a coordinating role in assisting with data acquisition, reviewed the manuscript for editing.

## FUNDING INFORMATION

This study was financed by the The Netherlands Organization for Health Research and Development (ZonMw) with grant number 104021004 and the Dutch Heart Foundation with grant number 2019B011.

## CONFLICT OF INTEREST STATEMENT

RvdL and RvE are cofounders, shareholders and board members of Cordys Analytics B.V., a spin‐off of the UMC Utrecht that has licensed several AI‐ECG algorithms. The UMC Utrecht receives royalties from Cordys Analytics for potential future revenues.

## Supporting information


Table S3.

## Data Availability

The datasets used in this study are not openly available due to privacy concerns.
