# Supplementary material for: Localization of accessory pathways in Wolff‐Parkinson‐white syndrome using ECG‐based multi‐task deep learning
Source: Eur J Clin Invest. 2025 Apr 7;55(Suppl 1):e14385. doi: 10.1111/eci.14385 (PMC11973841; doi:10.1111/eci.14385)
Supplement: Supplementary file 1 — Table S3. [file ECI-55-e14385-s001.docx]

Table 3: Results from the testing cohorts

|  | | **AUROC** | **Sens** | **Spec** | **PPV** | **NPV** | **Accuracy** |
| --- | --- | --- | --- | --- | --- | --- | --- |
| **Prospective UMCU test cohort** | | | | | | | |
| **WPW identification**, value [95%CI] | | 0.96 [0.89 - 1.00] | 0.57 [0.39 - 0.75] | 1.00 [1.00 - 1.00] | 0.39 [0.24 - 0.53] | 1.00 [1.00 - 1.00] | 1.00 [1.00 - 1.00] |
| **Location** | **Overall**, value [95%CI] | 0.98 [0.95 - 1.00] | 0.83 [0.70 - 0.93] | 0.81 [0.64 - 0.95] | 0.85 [0.71 - 0.95] | 0.87 [0.72 - 0.97] | 0.86 [0.74 - 0.96] |
|  | **Right**, value [95%CI] | 0.98 [0.00 - 1.00] | 1.00 [0.00 - 1.00] | 0.97 [0.00 - 1.00] | 0.50 [0.00 - 1.00] | 1.00 [0.00 - 1.00] | 0.97 [0.00 - 1.00] |
|  | **Septal**, value [95%CI] | 0.78 [0.61 - 0.93] | 0.60 [0.29 - 0.90] | 0.95 [0.83 - 1.00] | 0.86 [0.50 - 1.00] | 0.83 [0.65 - 0.96] | 0.83 [0.70 - 0.97] |
|  | **Left**, value [95%CI] | 0.84 [0.69 - 0.98] | 0.95 [0.82 - 1.00] | 0.73 [0.44 - 1.00] | 0.86 [0.68 - 1.00] | 0.89 [0.62 - 1.00] | 0.87 [0.73 - 0.97] |
| **Parahisian pathway**, value [95%CI] | | 0.95 [0.84 - 1.00] | 1.00 [1.00 - 1.00] | 0.92 [0.79 - 1.00] | 0.67 [0.20 - 1.00] | 1.00 [1.00 - 1.00] | 0.93 [0.80 - 1.00] |
| **Potential TSP prediction**, value [95%CI] | | 0.93 [0.82 - 1.00] | 0.86 [0.71 - 1.00] | 0.75 [0.38 - 1.00] | 0.90 [0.76 - 1.00] | 0.67 [0.33 - 1.00] | 0.83 [0.70 - 0.97] |
| **External MUI test cohort** | | | | | | | |
| **Location** | **Overall**, value [95%CI] | 0.87 [0.81 - 0.93] | 0.79 [0.69 - 0.90] | 0.83 [0.74 - 0.91] | 0.81 [0.65 - 0.90] | 0.86 [0.78 - 0.94] | 0.83 [0.74 - 0.92] |
|  | **Right**, value [95%CI] | 0.60 [0.50 - 0.83] | 0.20 [0.00 - 0.67] | 1.00 [1.00 - 1.00] | 1.00 [1.00 - 1.00] | 0.91 [0.83 - 0.98] | 0.92 [0.83 - 0.98] |
|  | **Septal**, value [95%CI] | 0.73 [0.61 - 0.85] | 0.83 [0.67 - 0.96] | 0.64 [0.43 - 0.82] | 0.68 [0.50 - 0.85] | 0.80 [0.61 - 0.95] | 0.73 [0.60 - 0.85] |
|  | **Left**, value [95%CI] | 0.80 [0.68 - 0.91] | 0.75 [0.54 - 0.93] | 0.86 [0.71 - 0.97] | 0.79 [0.58 - 0.95] | 0.83 [0.68 - 0.96] | 0.81 [0.69 - 0.92] |
| **Parahisian pathway**, value [95%CI] | | 0.83 [0.69 - 0.93] | 0.73 [0.43 - 1.00] | 0.81 [0.68 - 0.92] | 0.53 [0.27 - 0.79] | 0.91 [0.81 - 1.00] | 0.79 [0.67 - 0.90] |
| **Potential TSP prediction**, value [95%CI] | | 0.90 [0.81 - 0.97] | 1.00 [1.00 - 1.00] | 0.70 [0.52 - 0.87] | 0.72 [0.56 - 0.89] | 1.00 [1.00 - 1.00] | 0.83 [0.73 - 0.94] |

Table 3 Abbreviations: University Medical Centre Utrecht (UMCU), Medical University Innsbruck (MUI), Area under the Receiver Operating Characteristic curve (AUROC), Sensitivity (Sens), Specificity (Spec), positive predictive value (PPV), negative predictive value (NPV), Confidence interval (CI), Wolff-Parkinson-White syndrome (WPW), Transseptal puncture (TSP).
